# The Orphan Gene *dauerless* Regulates Dauer Development and Intraspecific Competition in Nematodes by Copy Number Variation

**DOI:** 10.1371/journal.pgen.1005146

**Published:** 2015-06-18

**Authors:** Melanie G. Mayer, Christian Rödelsperger, Hanh Witte, Metta Riebesell, Ralf J. Sommer

**Affiliations:** Department of Evolutionary Biology, Max Planck Institute for Developmental Biology, Tübingen, Germany; Stanford University Medical Center, UNITED STATES

## Abstract

Many nematodes form dauer larvae when exposed to unfavorable conditions, representing an example of phenotypic plasticity and a major survival and dispersal strategy. In *Caenorhabditis elegans*, the regulation of dauer induction is a model for pheromone, insulin, and steroid-hormone signaling. Recent studies in *Pristionchus pacificus* revealed substantial natural variation in various aspects of dauer development, i.e. pheromone production and sensing and dauer longevity and fitness. One intriguing example is a strain from Ohio, having extremely long-lived dauers associated with very high fitness and often forming the most dauers in response to other strains´ pheromones, including the reference strain from California. While such examples have been suggested to represent intraspecific competition among strains, the molecular mechanisms underlying these dauer-associated patterns are currently unknown. We generated recombinant-inbred-lines between the Californian and Ohioan strains and used quantitative-trait-loci analysis to investigate the molecular mechanism determining natural variation in dauer development. Surprisingly, we discovered that the orphan gene *dauerless* controls dauer formation by copy number variation. The Ohioan strain has one *dauerless* copy causing high dauer formation, whereas the Californian strain has two copies, resulting in strongly reduced dauer formation. Transgenic animals expressing multiple copies do not form dauers. *dauerless* is exclusively expressed in CAN neurons, and both CAN ablation and *dauerless* mutations increase dauer formation. Strikingly, *dauerless* underwent several duplications and acts in parallel or downstream of steroid-hormone signaling but upstream of the nuclear-hormone-receptor *daf-12*. We identified the novel or fast-evolving gene *dauerless* as inhibitor of dauer development. Our findings reveal the importance of gene duplications and copy number variations for orphan gene function and suggest *daf-12* as major target for dauer regulation. We discuss the consequences of the novel *vs.* fast-evolving nature of orphans for the evolution of developmental networks and their role in natural variation and intraspecific competition.

## Author Summary

The nematode dauer stage represents the major survival and dispersal strategy, and had a remarkable impact in the evolutionary and ecological success of nematodes. Our recent work in *Pristionchus pacificus* revealed substantial natural variation in various aspects of dauer development, i.e. pheromone production and sensing and dauer longevity and fitness, including a strain from Ohio with extremely long-lived dauers, very high fitness and high dauer formation in response to other strains´ pheromones. However, the molecular mechanisms associated with natural variation in dauer development are currently unknown. Using quantitative-trait-loci analysis, we discovered that the orphan gene *dauerless* controls dauer formation by copy number variation. Strains with one *dauerless* copy show high dauer formation, whereas strains with two copies have strongly reduced dauer formation. Transgenic animals expressing multiple copies do not form dauers. *dauerless* is exclusively expressed in CAN neurons, and both CAN ablation and *dauerless* mutations increase dauer formation. Strikingly, *dauerless* underwent several duplications and acts in parallel or downstream of steroid-hormone signaling but upstream of the nuclear-hormone-receptor *daf-12*. Our findings reveal the importance of gene duplications and copy number variations for orphan gene function and suggest *daf-12* as major target for dauer regulation.

## Introduction

**Fig 1:**
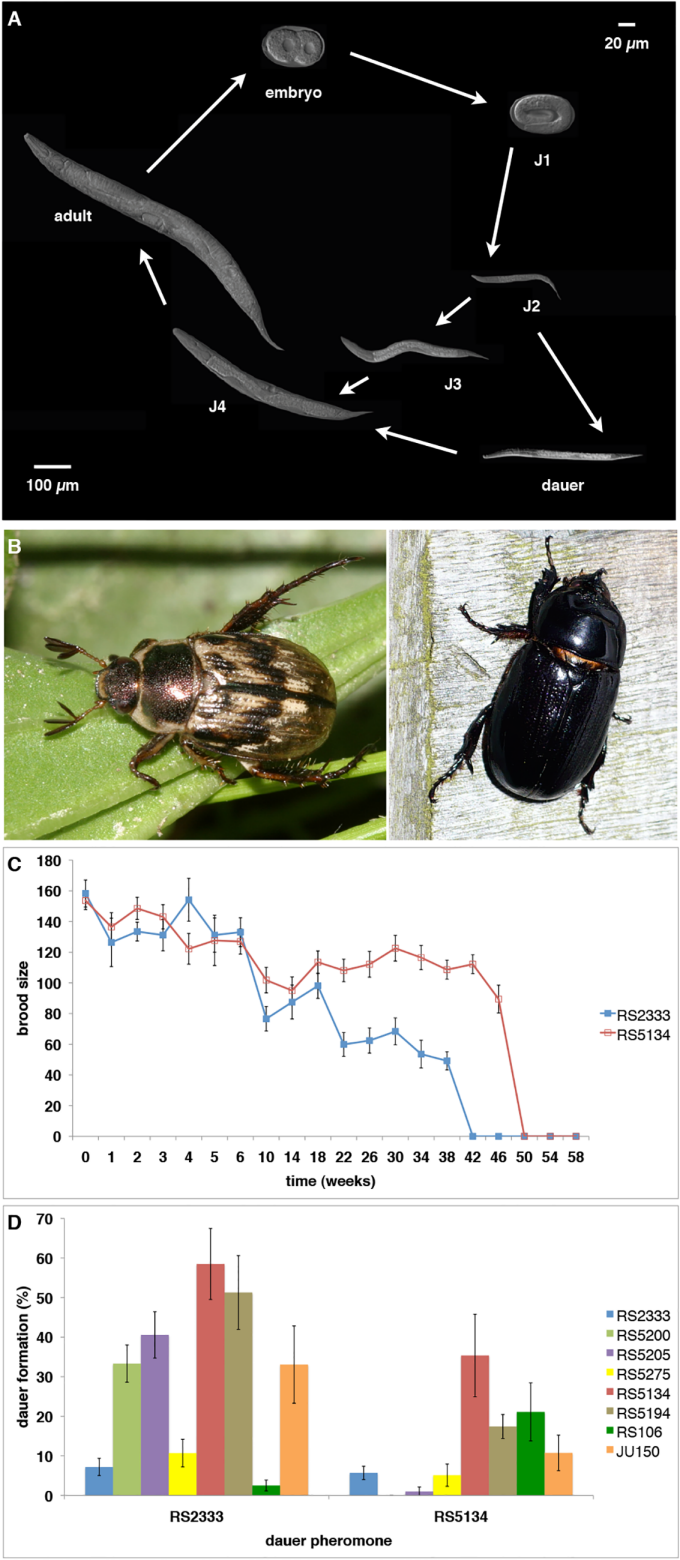
*P*. *pacificus* dauer formation and natural variation. (**A**) *P*. *pacificus* life cycle. (**B**) The scarab beetles *Exomala orientalis* (left) and *Oryctes borbonicus* (right) are two natural hosts of *P*. *pacificus* in Japan and La Réunion Island, respectively. (**C**) Natural variation in dauer longevity and fitness as measured by brood size after recovery from cold storage (data from [13]). The strain RS5134 from Ohio represents the most long-lived strain of *P*. *pacificus* and has a relatively high brood size of approximately 100 progeny even after a cold storage of 46 weeks. (**D**) Cross-preference of eight strains in response to RS2333 and RS5134 dauer pheromone (data from [13]).

Phenotypic (developmental) plasticity describes the ability of an individual organism to develop distinct phenotypes from the same genotype. Besides numerous examples in plants and insects, nematode dauer development represents one key example of phenotypic plasticity (Fig 1) [1]. The nematode model organisms *Caenorhabditis elegans* and *Pristionchus pacificus* undergo direct development through four larval stages under favorable environmental conditions, reaching adulthood in as little as three days under standard laboratory conditions (20°C) (Fig 1A). In contrast, unfavorable conditions, such as high temperature, low food availability, and high population density, result in the formation of long-lived dauer larvae [2]. Dauer larvae are resistant to many environmental stresses and show several morphological and behavioral adaptations. They have a closed mouth and a thick cuticle, enabling survival under harsh conditions. In addition, many dauer larvae show a nictation or waving behavior (Winkverhalten), which is usually considered to represent a dispersal strategy, allowing dauer larvae to attach to and disperse with various invertebrates. For example, *P*. *pacificus* is associated with scarab beetles in the wild and shows a necromenic association with its beetle hosts (Fig 1B) [3]. On the living beetle, nematodes are exclusively found in the dauer stage, and they resume development only after the beetle´s natural death by feeding on developing microbes on the carcass [4]. Therefore, the nematode dauer stage is usually considered to represent the most important dispersal and survival strategy that has contributed enormously to the evolutionary success of this taxon [5].

In *C*. *elegans*, dauer development is regulated by a complex genetic network involving pheromone, insulin, TGF-β, and endocrine signaling [6]. The dauer pheromone consists of a modular library of small-molecule signals, the ascarosides, which induce the formation of dauer larvae [7]. Several signaling pathways, including insulin, TGF-β, and cGMP signaling, have been shown to transmit various environmental signals and are thought to converge on an endocrine signaling module. Endocrine signaling consists of the nuclear hormone receptor DAF-12 that acts as a developmental switch [8]. In its ligand-free state, DAF-12 induces dauer formation and consistently, *daf-12* loss-of-function mutants are dauer-formation-defective (Daf-d). In contrast, in the presence of the DAF-12 ligand, dauer formation is inhibited. Several derivatives of the steroid hormone dafachronic acid (DA) were shown to act as DAF-12 ligands, including ∆7-DA, ∆4-DA, and ∆1, ∆7-DA [9,10].

Besides these detailed genetic and molecular studies that made dauer formation an important model in biomedical research, various studies in *P*. *pacificus* have established nematode dauer formation as a model system for investigating natural variation and its consequences on the evolutionary ecology of the organism. First, Bose and coworkers (2014) showed substantial natural variation in pheromone signaling [11]. In *P*. *pacificus*, dauer pheromones consist of a blend of ascarosides and paratosides with chemically very diverse building blocks from all major metabolic pathways [7,12]. The comparison of the composition of the pheromones of six natural isolates of *P*. *pacificus* revealed tremendous variation in pheromone composition, even among three strains from the same habitat on La Réunion Island in the Indian Ocean. Second, when exposed to individual pheromone components different strains showed enormous variation in their dauer formation response with little correlation between pheromone production and pheromone sensing [11]. These results have been interpreted as cross-preference and indication for intraspecific competition, a phenomenon to be described below. Third, dauer larvae were also shown to differ in their survival properties [13]. Specifically, we showed that dauer larvae of eight tested *P*. *pacificus* wild isolates survived under standardized laboratory conditions for 25 to 50 weeks. The strain RS5134 isolated from a scarab beetle in Ohio (USA) showed one of the most extreme survival rates of 50 weeks in distilled water at 8°C (Fig 1C). Finally, these strains not only survived but were also able to reproduce after dauer recovery as indicated by brood size tests [13]. Again, variation in fitness was observed with the strain from RS5134/Ohio producing approximately 100 progeny after 46 weeks in the dauer stage (Fig 1C). Together, these studies support the notion that all tested aspects of dauer induction and exit show substantial natural variation.

One of the most striking findings of these natural variation studies was the lack of correlation between small-molecule production and sensing. The analysis of pheromone extracts of 16 *P*. *pacificus* wild isolates had already indicated that most dauer pheromones induce higher dauer formation in other *P*. *pacificus* genotypes rather than in their own (Fig 1D) [13]. For example, the strain RS5134 from Ohio formed more dauers in response to the pheromone of the wild-type strain RS2333 from California than in response to its own pheromone. This response pattern, which was observed in 13 of 16 tested strains, has been described as “cross-preference” (Fig 1D) [13]. Follow-up analysis indicated that the strains RS2333/California and RS5134/Ohio differ in the exact composition of their dauer pheromones [11]. Inspired by these surprising results on natural variation in dauer pheromone production and sensing in *P*. *pacificus*, a novel assay was established to analyze if natural isolates can compete for dauer induction [11]. While our competition experiments support intraspecific competition in nematode dauer formation, the underlying molecular mechanisms and the potential ecological consequences of these results remain largely unknown. In evolutionary terms, intraspecific competition has been suggested to be associated with evolutionary arms races and to represent a strong selective force driving the divergence among populations [14,15]. Indeed, studies in bacteria have provided detailed insights into competitive interactions often involving toxin-antitoxin systems [16], but little is known about the genetic mechanisms underlying intraspecific competition in animals.

Here, we investigate the molecular mechanisms underlying the observed cross-preference and competition between RS2333/California and RS5134/Ohio. Using a recombinant-inbred-line (RIL) and quantitative-trait-loci (QTL) approach, we made the surprising finding that intraspecific competition relies on an orphan gene, *dauerless*, that acts by copy number variation. *dauerless* is exclusively expressed in CAN neurons, and animals in which the CAN neurons have been ablated, as well as *dauerless* deletion mutants generated by the CRISPR/Cas9 system, show increased dauer formation. Finally, epistasis analysis indicates that *dauerless* acts downstream or in parallel of steroid-hormone signaling but upstream of *daf-12*. Our findings reveal the importance of gene duplications and dosage effects and indicate that novel or fast-evolving genes can have key functions in developmental regulatory networks.

## Results

### RS2333/California and RS5134/Ohio show cross-preference of dauer pheromones

The molecular mechanisms underlying cross-preference and intraspecific competition among nematode populations can best be investigated by RIL and QTL approaches. We selected the two strains RS2333/California and RS5134/Ohio for molecular investigations because of their large differences in dauer induction and because RS5134/Ohio has the highest dauer formation in response to the pheromone of RS2333/California out of eight tested strains [13]. Specifically, RS5134/Ohio shows nearly 60% dauer formation in response to the RS2333/California pheromone, but only 35% dauer formation in response to its own pheromone (Fisher's exact test, P<0.006) (Fig 1D). In contrast, RS2333/California has a low dauer formation phenotype in response to its own and the RS5134/Ohio pheromone (Fig 1D), indicating that the California—Ohio pair has robust phenotypic differences that allow QTL analysis.

**Fig 2:**
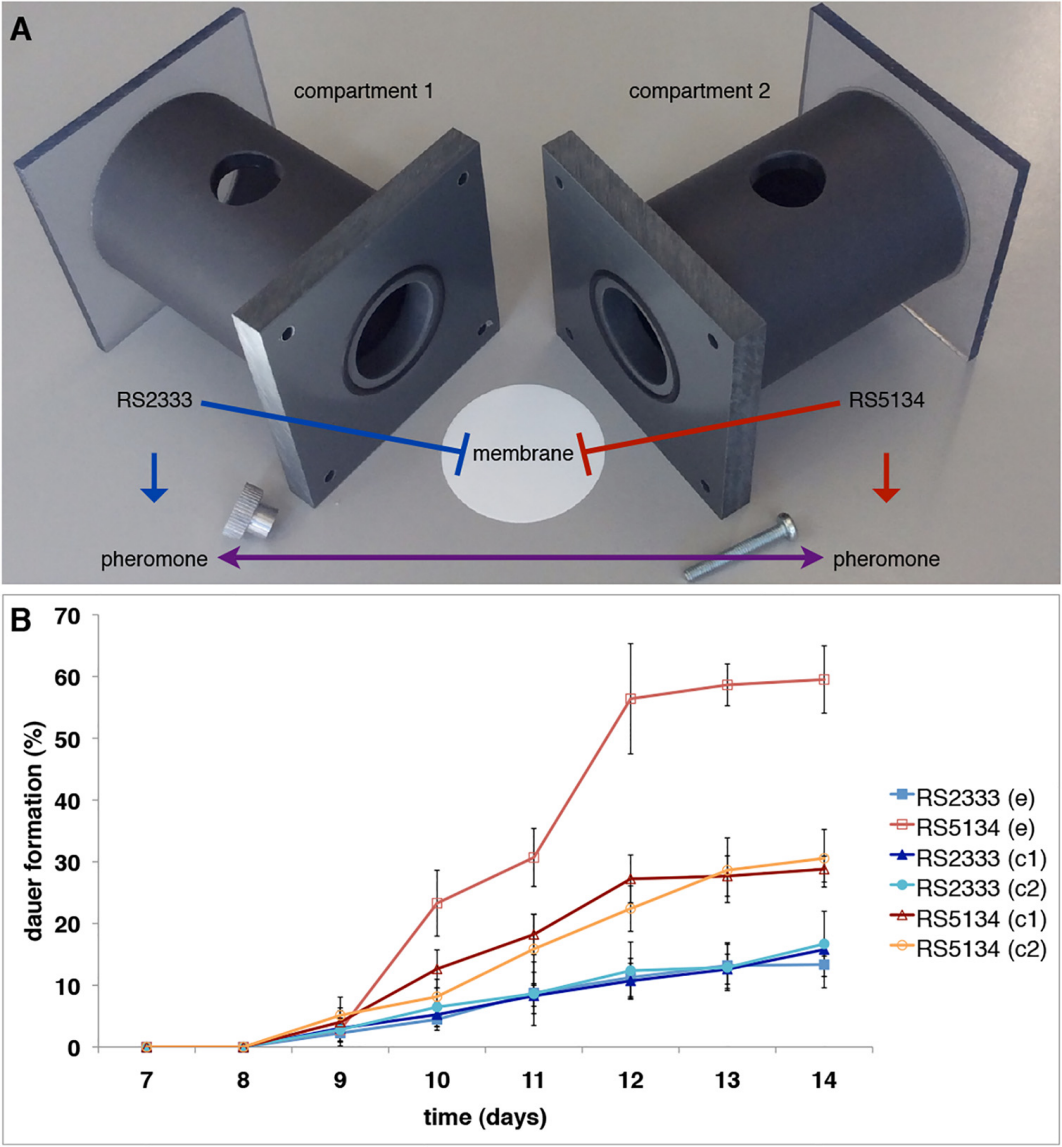
Ussing chamber setup and competition assay. **(A)** Ussing chamber competition assay setup. (B) Intraspecific competition between RS2333 and RS5134 observed over time. In control experiments (c) both compartments of the Ussing chamber are filled with the same strain, whereas in the competition experiment (e) one compartment contains RS2333 and the other RS5134. Values are mean dauer formation of three replicates. Error bars represent 95% confidence intervals.

The cross-preference between RS2333/California and RS5134/Ohio was originally observed in dauer pheromone assays (Fig 1D) [13]. To test for the existence of intraspecific competition between RS2333/California and RS5134/Ohio we performed competition assays in Ussing chambers (Fig 2A) and measured dauer formation over time (Fig 2B). Starting at days 10 and 11, RS5134/Ohio formed more dauers when exposed to the RS2333/California pheromone than when only exposed to its own pheromone in the control (Fisher's exact test, P<0.003) (Fig 2B). In contrast, RS2333/California showed approximately the same dauer formation phenotype when exposed to the RS5134/Ohio pheromone as in the control experiment after the same amount of time (Fig 2B). In the following, up to day 14 (Fig 2B), RS2333/California dauer formation stayed low at approximately 15%. In contrast, RS5134/Ohio showed increased dauer formation reaching 30% in the control, but 60% when exposed to the RS2333/California pheromone (Fisher's exact test, P<0.0001) (Fig 2B). These results indicate that cross-preference of dauer pheromones leads to a higher dauer formation phenotype in RS5134/Ohio when exposed to the RS2333/California pheromone possibly indicating that strains can compete for dauer entry.

### Genetic basis of cross-preference between RS2333/California and RS5134/Ohio

**Fig 3:**
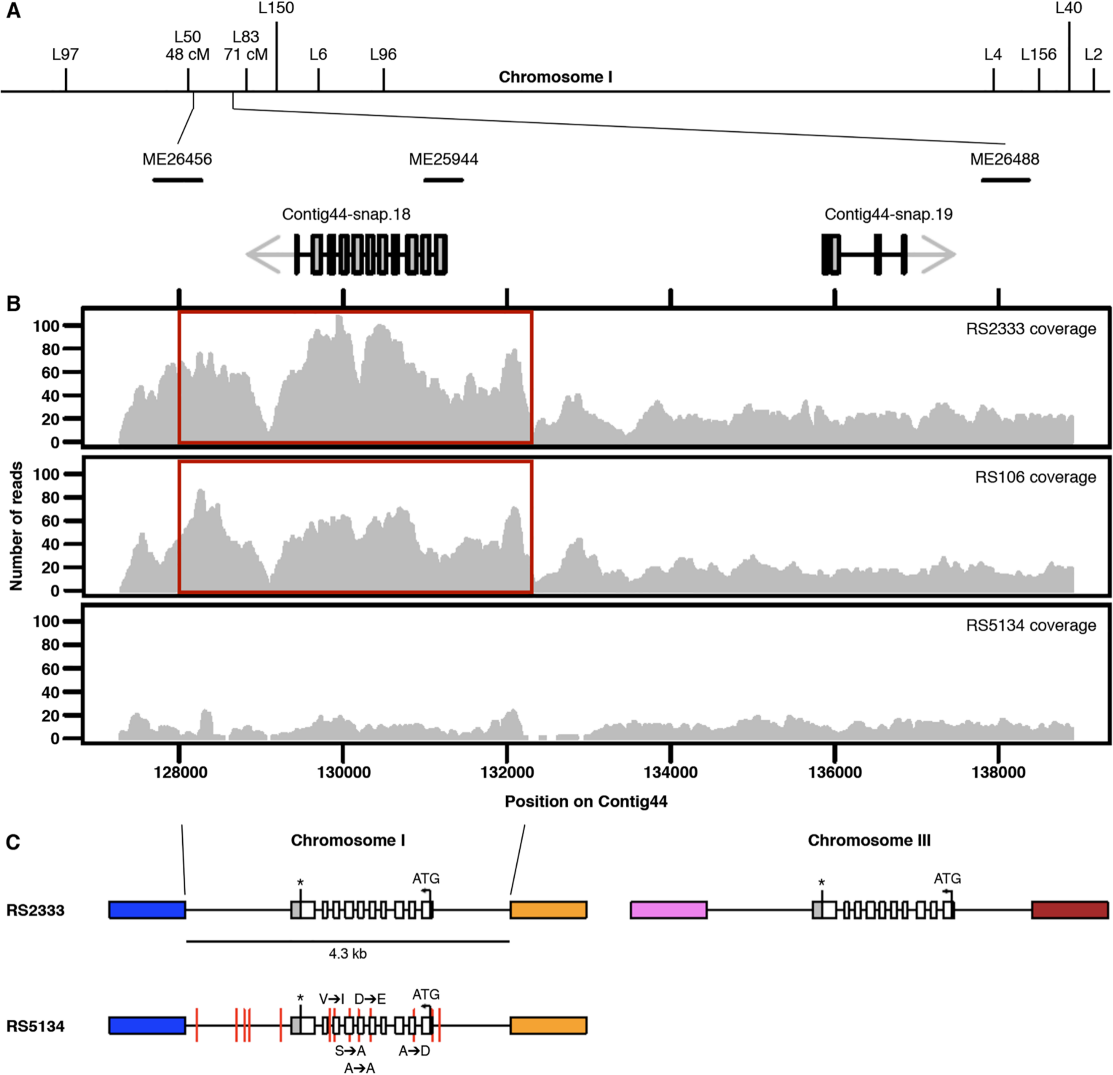
Genomic characterization. (**A**) Genetic map of chromosome I. The chromosomal interval associated with the QTL ME25944 contains two gene predictions. (**B**) RS2333, RS106, and RS5134 resequencing coverage. The 4.3 kb duplicated region is highlighted in red. (**C**) Copy numbers and organization of Contig44-snap.18 in RS2333 and RS5134. Colored blocks indicate unique sequences flanking the 4.3 kb duplicated region. Red vertical lines represent the 13 SNPs between RS2333 and RS5134.

To elucidate the molecular mechanisms underlying intraspecific competition, we generated 911 RILs of RS2333/California and RS5134/Ohio (S1A Fig). For each RIL, we determined the dauer formation phenotype in response to both parental pheromones (see Materials and Methods). Next, we selected 136 RILs, covering all phenotypic classes (high, intermediate and low dauer formation phenotypes) for genotyping with simple sequence length and conformation polymorphism markers [17]. Using QTL mapping, we indentified six QTL peaks with significantly high logorithm-of-odds (LOD) scores (S1B Fig). Fine mapping enabled us to narrow down the QTL peak associated with the marker ME25944 on chromosome I to a 10 kb region based on the high recombination frequency in this interval of the *P*. *pacificus* genome (Fig 3). This 10 kb region contains only two gene predictions, the orphan gene Contig44-snap.18 and the globin-like gene Contig44-snap.19 (Fig 3A). Comparison of expression levels by RNA-seq between RS2333/California and RS5134/Ohio showed a strong upregulation of Contig44-snap.18 in RS2333/California (FDR<0.05), which makes Contig44-snap.18 the prime candidate for the causative gene within the QTL peak. Contig44-snap.18 contains 10 exons with a conceptual translation into a polypetide of 314 amino acids (Fig 3A). There is no sequence similarity at the DNA and protein level of Contig44-snap.18 to sequences outside the genera *Pristionchus* and *Parapristionchus* (S2 Fig). Also, we did not find any signal peptide or other known sequence motifs using the programs SignalP4.1 and prosite release 20.111. Thus, Contig44-snap.18 represents a true orphan (pioneer) gene (for details see below). Orphan genes are common in *P*. *pacificus* and other nematodes [18,19], and a large number of the *P*. *pacificus* orphan genes have been shown to be expressed in transcriptomics and proteomics studies [20,21].

### Copy number variation of the orphan gene Contig44-snap.18

Further investigation of Contig44-snap.18 suggests copy number variation (CNV) at this locus between RS2333/California and RS5134/Ohio. In the RS2333/California genome assembly, Contig44-snap.18 is located in a 4.3 kb region for which the coverage is approximately twice as high as for the adjacent regions (Fig 3B). This difference in coverage does not exist in RS5134/Ohio, suggesting that the region was duplicated in RS2333/California but not in RS5134/Ohio (Fig 3B). Additional support for a local and recent duplication event comes from the re-sequencing project of 104 wild isolates of *P*. *pacificus* [22]. The strain RS106 from Poland, which represents the strain most closely related to RS2333/California, is the only other strain with a difference in coverage of Contig44-snap.18 (Fig 3B).

Using inverse PCR, we determined the location of the duplicated region on chromosome III of RS2333/California (Fig 3C). The second copy of Contig44-snap.18 on chromosome III is identical to Contig44-snap.18 on chromosome I over a 4.3 kb interval, as expected for a recent duplication event. This includes 1.1 kb of upstream and 1.5 kb of downstream sequences (Fig 3C). In contrast, the Contig44-snap.18 genes on chromosome I of RS2333/California and RS5134/Ohio contain a total of 5 single nucleotide polymorphisms (SNPs) in the 1.7 kb open reading frame and a total of 13 SNPs in the complete 4.3 kb region (Fig 3C). Interestingly, the second copy of Contig44-snap.18 gene on chromosome III of RS2333/California is also associated with one of our QTL peaks. However, due to the much lower recombination frequency in this area of the *P*. *pacificus* genome, our attempts at finemapping of this QTL were unsuccessful. Together, these findings further support the role of the orphan gene Contig44-snap.18 as prime candidate for the QTL on chromosome I and let us hypothersize that the observed phenotypic differences between the two strains are caused by a recent CNV.

### Expression of multiple copies of Contig44-snap.18 results in a dauerless phenotype

The CNV hypothesis for Contig44-snap.18 suggests that the presence of one gene copy results in high dauer formation, whereas two gene copies cause low dauer formation in response to dauer pheromones. To test this hypothesis and to determine if Contig44-snap.18 indeed plays a role in dauer formation, we generated transgenic lines that carry multiple copies of Contig44-snap.18. According to the CNV hypothesis, a further increase in Contig44-snap.18 copies should further decrease dauer formation after exposure to dauer pheromone. We injected a 9.5 kb genomic construct of RS2333/California or RS5134/Ohio, consisting of a 3.3 kb upstream (promoter) region, the 1.7 kb Contig44-snap.18 open reading frame, and a 4.5 kb downstream region, into RS2333/California or RS5134/Ohio animals. We generated a total of five transgenic lines carrying the RS2333/California version of Contig44-snap.18 in either the RS2333/California or RS5134/Ohio genetic background and the RS5134/Ohio version of Contig44-snap.18 in RS2333/California (Table 1). Strikingly, transgenic animals of all five lines do not form dauers at all (Table 1 c1,d1,e1,f1,g1). In contrast, non-transgenic nematodes that have lost the transgenic array show the wild-type RS2333/California or RS5134/Ohio dauer formation phenotype (Table 1 c2,d2,e2,f2,g2). Furthermore, in a transgenic control line, which only expresses the red-fluorescent-protein (RFP) injection marker, both transgenic and non-transgenic animals form dauers at wild-type frequencies (Table 1 h1,h2). These results support the CNV hypothesis for Contig44-snap.18: One copy results in the high dauer formation phenotype of RS5134/Ohio, two copies lead to the low dauer formation phenotype of RS2333/California, and multiple copies eliminate dauer formation in transgenic animals. Since the expression of multiple copies of Contig44-snap.18 completely inhibits dauer formation, we named the gene *dauerless*, *dau-1*.*1* for the copy on chromosome I and *dau-1*.*2* for the copy on chromsome III.

**Table 1:** Dauer formation of transgenic lines, CAN ablated animals, and *dau-1* mutants in response to RS2333 and RS5134 pheromone.

	strain or line	chol.^a^	RS2333 pheromone		RS5134 pheromone	
			dauer (%)^b^	*p* value^c^	dauer (%)^b^	p value^c^
**a**	RS2333 (CA)	+	11		5	
**b**	RS5134 (OH)	+	68		39	
**c1**	RS5134;Ex[*dau-1*.*1* ^CA^] RFP^+^	+	0	<0.0001 **b**	0	<0.0001 **b**
**c2**	RS5134;Ex[*dau-1*.*1* ^CA^] RFP^-^	+	62	0.6001 **b**	29	0.4710 **b**
**d1**	RS2333;Ex[*dau-1*.*1* ^CA^] line1 RFP^+^	+	0	0.0009 **a**	0	0.0827 **a**
**d2**	RS2333;Ex[*dau-1*.*1* ^CA^] line1 RFP^-^	+	11	1 **a**	7	0.5883 **a**
**e1**	RS2333;Ex[*dau-1*.*1* ^CA^] line2 RFP^+^	+	0	0.0101 **a**	0	0.1871 **a**
**e2**	RS2333;Ex[*dau-1*.*1* ^CA^] line2 RFP^-^	+	6	0.7355 **a**	7	0.6565 **a**
**f1**	RS2333;Ex[*dau-1*.*1* ^CA^] line3 RFP^+^	+	0	0.0046 **a**	0	0.0930 **a**
**f2**	RS2333;Ex[*dau-1*.*1* ^CA^] line3 RFP^-^	+	10	1 **a**	6	1 **a**
**g1**	RS2333;Ex[*dau-1*.*1* ^OH^] RFP^+^	+	0	0.0720 **a**	0	0.3369 **a**
**g2**	RS2333;Ex[*dau-1*.*1* ^OH^] RFP^-^	+	12	1 **a**	4	1 **a**
**h1**	RS2333;Ex[*egl-20*::RFP] RFP^+^	+	10	1 **a**	6	0.7455 **a**
**h2**	RS2333;Ex[*egl-20*::RFP] RFP^-^	+	9	0.7799 **a**	7	0.4486 **a**
**i**	RS2333 (CA)	-	22	0.0472 **a**	14	0.0418 **a**
**j**	RS5134 (OH)	-	87	0.0002 **b**	53	0.0215 **b**
**k1**	RS5134;Ex[*dau-1*.*1* ^CA^] RFP^+^	-	0	1 **c1**	0	1 **c1**
**k2**	RS5134;Ex[*dau-1*.*1* ^CA^] RFP^-^	-	82	0.4170 **c2**	55	0.2082 **c2**
**l1**	RS2333;Ex[*dau-1*.*1* ^CA^] line1 RFP^+^	-	0	1 **d1**	0	1 **d1**
**l2**	RS2333;Ex[*dau-1*.*1* ^CA^] line1 RFP^-^	-	16	1 **d2**	20	0.3650 **d2**
**m1**	RS2333;Ex[*dau-1*.*1* ^CA^] line2 RFP^+^	-	0	1 **e1**	0	1 **e1**
**m2**	RS2333;Ex[*dau-1*.*1* ^CA^] line2 RFP^-^	-	20	0.3364 **e2**	10	1 **e2**
**n1**	RS2333;Ex[*dau-1*.*1* ^CA^] line3 RFP^+^	-	0	1 **f1**	0	1 **f1**
**n2**	RS2333;Ex[*dau-1*.*1* ^CA^] line3 RFP^-^	-	19	1 **f2**	15	0.6026 **f2**
**o1**	RS2333;Ex[*dau-1*.*1* ^OH^] RFP^+^	-	0	1 **g1**	0	1 **g1**
**o2**	RS2333;Ex[*dau-1*.*1* ^OH^] RFP^-^	-	22	0.3255 **g2**	13	0.3232 **g2**
**p1**	RS2333;Ex[*egl-20*::RFP] RFP^+^	-	21	0.1506 **h1**	13	0.2274 **h1**
**p2**	RS2333;Ex[*egl-20*::RFP] RFP^-^	-	23	0.1176 **h2**	17	0.3320 **h2**
**q**	RS2333	-	21		15	
**r**	RS2333 CAN^-^	-	94	<0.0001 **q**	75	<0.0001 **q**
**s**	RS5134	-	79		58	
**t**	RS5134 CAN^-^	-	88	0.4149 **s**	83	0.0156 **s**
**u**	RS2333;Ex[*dau-1*.*1*::RFP]	-	0		0	
**v**	RS2333;Ex[*dau-1*.*1*::RFP] CAN^-^	-	95	<0.0001 **u**	78	<0.0001 **u**
**w1**	*dau-1*.*1(tu490)*	-	83	<0.0001 **q**	54	0.0001 **q**
				0.7953 **s**		0.8405 **s**
**w2**	*dau-1*.*1(tu491)*	-	79	<0.0001 **q**	59	<0.0001 **q**
				1 **s**		1 **s**
**w3**	*dau-1*.*2(tu492)*	-	78	<0.0001 **q**	55	<0.0001 **q**
				1 **s**		1 **s**
**x1**	*dau-1*.*1(tu490);dau-1*.*2(tu492)*	-	91	0.7150 **r**	77	0.8153 **r**
				0.5536 **w1**		0.0196 **w1**
				0.2623 **w2**		0.0828 **w2**
				0.1714 **w3**		0.0327 **w3**
**x2**	*dau-1*.*1(tu490);dau-1*.*2(tu492)*+DA	-	0	<0.0001 **x1**	0	<0.0001 **x1**
**y**	*daf-12(tu389)*	-	0		0	
**z**	*tu389;tu490;tu492*	-	0	<0.0001 **x1**	0	<0.0001 **x1**

^a^presence (+) or absence (-) of cholesterol in NGM agar plates.

^b^mean dauer formation of three biological replicates.

^c^ *p* value of Fisher's exact test.

### *dau-1* is exclusively expressed in CAN neurons

**Fig 4:**
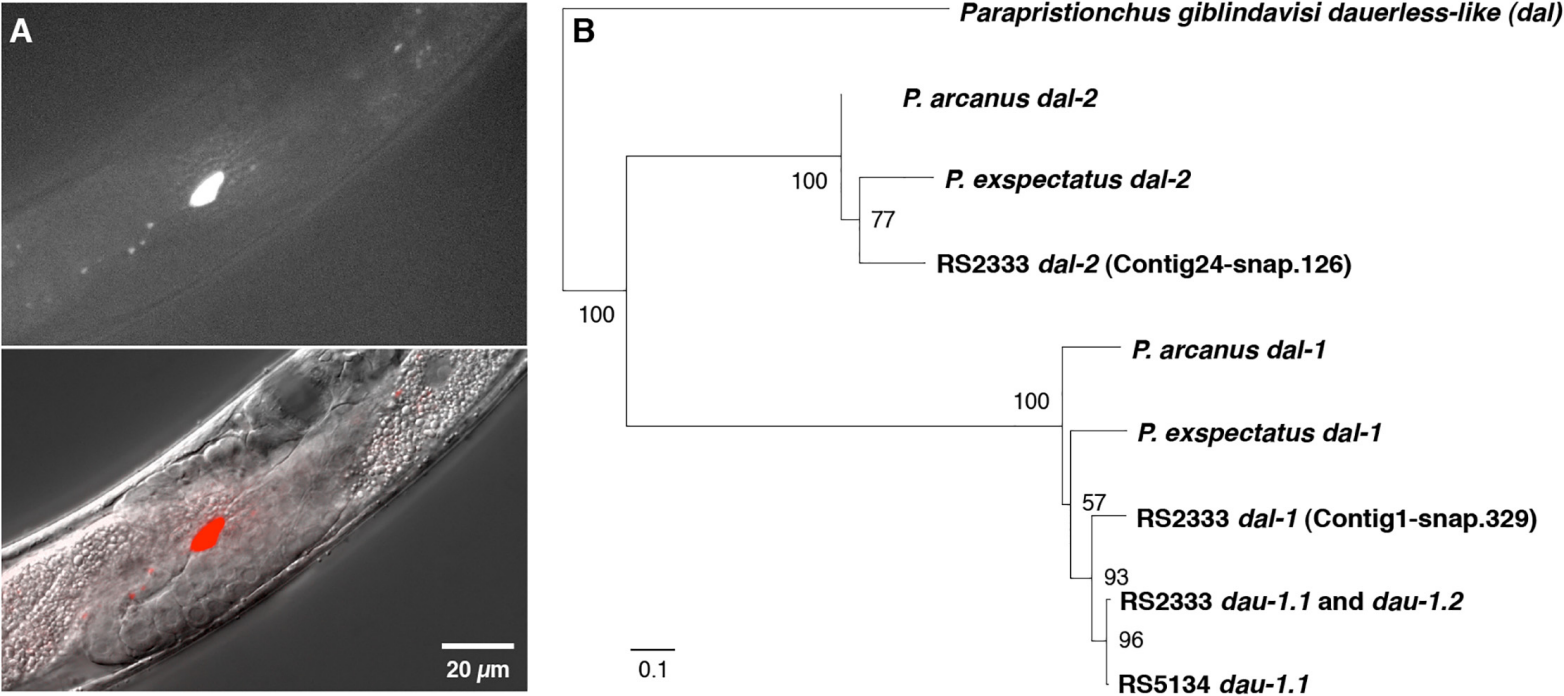
*dau-1* expression and phylogeny. (**A**) *dau-1* is expressed in CAN neurons. Image shows the cell body of the left CAN neuron. Neuronal processes are not visible in this focal plane. (**B**) *dau-1* phylogeny indicates several independent duplications after the speciation event leading to *P*. *pacificus*.

Given the absence of any sequence similarity of *dau-1* to genes in other organisms, we next wanted to determine the expression pattern of *dau-1* to obtain additional insight into its function. We generated three independent transgenic lines by injecting an RS2333/California translational reporter construct, containing a 4.7 kb promoter region and the *dau-1* open reading frame driving RFP expression, into RS2333/California animals. Surprisingly, we found that *dau-1* is exclusively expressed in the CAN neurons, which are a pair of neurons in the mid-body region born in late embryogenesis (Fig 4A). Specifically, we observed RFP expression in CAN neurons in the J2, J3, and J4 stages of all three transgenic lines. *dau-1* expression in CAN neurons suggests a previously unknown role for these neurons in *P*. *pacificus* dauer formation. In *C*. *elegans*, little is known about the function of CAN neurons other than that they are essential for survival. Specifically, *C*. *elegans* animals in which CAN neurons have been ablated die within 24 hours.

### Ablation of CAN neurons causes highly increased dauer formation

**Fig 5:**
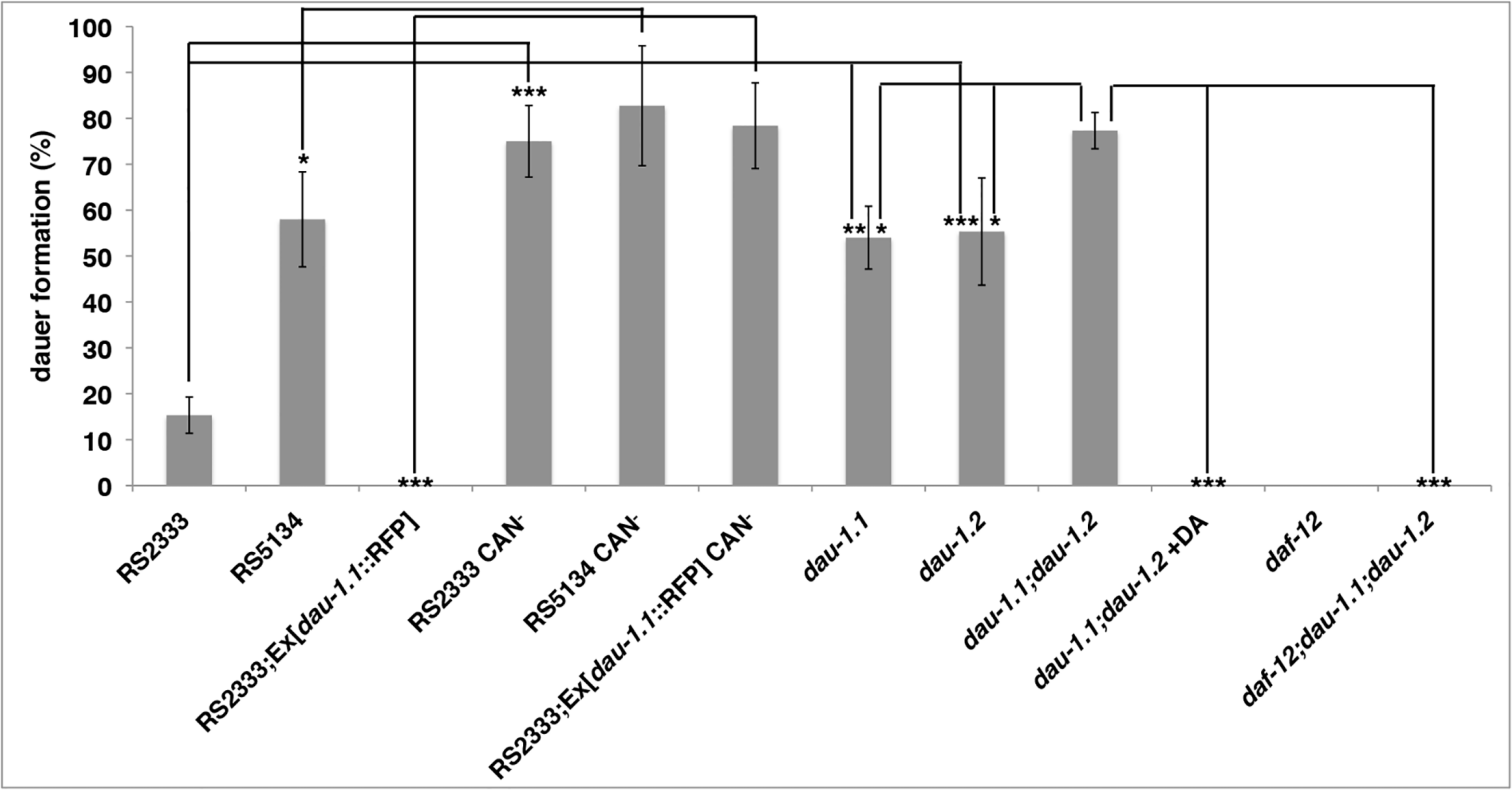
*dau-1* genetic and physical ablation. Dauer formation (mean of three replicates) of CAN ablated and *dau-1* mutant animals in response to RS5134 pheromone. Error bars represent 95% confidence intervals. *P<0.05, **P<0.0005, and ***P<0.0001 (Fisher's exact test). See Table 1 for details.

In contrast to *C*. *elegans*, CAN ablation in *P*. *pacificus* is viable. Therefore, to analyze the function of CAN neurons in dauer formation, we ablated the CAN neurons after hatching and performed dauer pheromone assays with J2 larvae (Fig 5; Table 1 q-v). Surprisingly, after CAN ablation RS2333/California animals show 94% and 75% dauer formation in response to RS2333/California and RS5134/Ohio pheromone, respectively (Fig 5; Table 1 r)). This dauer formation phenotype is not only higher than the wild type RS2333/California response but also higher than the wild type RS5134/Ohio response, suggesting that CAN neurons are part of a network repressing dauer formation. Similarly, CAN ablation in RS5134/Ohio resulted in extremely high dauer formation in response to both pheromones (Fig 5; Table 1 t). Most surprisingly however, a transgenic line expressing the *dau-1*.*1*::RFP reporter construct also showed an extremely high dauer formation phenotype in pheromone response, indicating that *dau-1* expression in CAN neurons is necessary for the *dau-1*-mediated inhibition of dauer formation (Fig 5; Table 1 v). The observed CAN ablation phenotype is consistent with the CNV hypothesis of *dau-1*: Expression of an increased number of *dau-1* copies reduces dauer formation. In contrast, eliminating *dau-1* by CAN ablation increases dauer response above wild type levels, suggesting that *dau-1* represents a strong suppressor of dauer development.

### *dau-1* RS2333/California single mutants mimic the RS5134/Ohio phenotype

To further support the idea that *dau-1* is a suppressor of dauer formation, we tested if the specific elimination of one of the two copies of *dau-1* in RS2333/California would increase the dauer formation phenotype to wild type RS5134/Ohio levels. We generated deletion mutants in the RS2333/California background using the CRISPR/Cas9 system [23] and obtained two lines (*tu490* and *tu491*) with a deletion in *dau-1*.*1* and one line (*tu492*) with a deletion in *dau-1*.*2*. All three mutant lines form more dauers than wild type RS2333/California (Fig 5; Table 1 w1,w2,w3). Furthermore, the mutant dauer formation phenotype mimics the wild type RS5134/Ohio phenotype in response to both dauer pheromones. Thus, eliminating one *dau-1* copy in RS2333/California is sufficient to increase the level of dauer formation to the RS5134/Ohio phenotype.

### *dau-1* RS2333/California double mutant mimics the CAN ablation phenotype

Next, we wanted to know if a *dau-1* double mutant in the RS2333/California background would further increase the dauer formation phenotype to the level observed after CAN ablation. We generated a double mutant by crossing the single mutant lines *dau-1*.*1(tu490)* and *dau-1*.*2(tu492)*. Indeed, the resulting double mutant *dau-1*.*1(tu490)*; *dau-1*.*2(tu492)* showed a further increase in dauer formation after treatment with both pheromones (Fig 5; Table 1 x1). Specifically, after treatment with the RS2333/California pheromone double mutant animals formed 91% dauers as compared to 94% dauer formation after CAN ablation (Fig 5; Table 1 r, x1). Similarly, in response to RS5134/Ohio pheromone, double mutant and CAN ablated animals formed approximately 80% dauers, whereas both single mutants formed significantly fewer dauers after the same treatment (Fig 5; Table 1 r, x1). Note, that statistically significant differences were only observed after treatment with the RS5134/Ohio pheromone, most likely due to the lower baseline of dauer formation in comparison to the RS2333/California pheromone. Together, these findings provide final support for the CNV hypothesis and indicate that the role of the CAN neurons in dauer formation is exclusively regulated by the *dau-1* genes.

### *daf-12* is epistatic to *dau-1*

To determine if *dau-1* acts upstream or downstream of endocrine signaling, we performed epistasis analysis with a *daf-12* mutant and cholesterol depletion experiments. Dauer formation in *P*. *pacificus* is known to involve cholesterol-derived steroid hormones and therefore, we made use of the fact that dauer induction by pheromones can be enhanced by cholesterol depletion [24]. In control experiments, wild type RS2333/California and RS5134/Ohio animals showed increased dauer formation in the absence of cholesterol in response to both pheromones (Table 1 i, j). However, when we tested the *dau-1* transgenic lines in dauer pheromone assays using agar plates without cholesterol, all transgenic animals still showed 0% dauer formation (Table 1 k1, l1, m1, n1, o1). In contrast, the non-transgenic nematodes that have lost the transgenic array and the transgenic control line show increased dauer formation (Table 1 k2, l2, m2, n2, o2, p1, p2). Thus, expression of multiple copies of *dau-1* completely inhibits dauer formation even after cholesterol depletion, indicating that *dau-1* acts downstream of or in parallel to steroid-hormone signaling.

Next, we used epistasis analysis between *dau-1* and the nuclear hormone receptor *daf-12* by generating a *dau-1*.*1(tu490);dau-1*.*2(tu492);daf-12(tu389)* triple mutant. Interestingly, triple mutant animals show no dauer formation after pheromone treatment and thus, mimic the *daf-12* single mutant phenotype (Fig 5; Table 1 y, z). This finding indicates that *daf-12* is epistatic to *dau-1*. Similarly, application of ∆7-DA does inhibit dauer formation in the *dau-1*.*1(tu490);dau-1*.*2(tu492)* double mutant (Fig 5; Table 1 x2). Together with the cholesterol depletion experiments, these results indicate that *dau-1* acts downstream or in parallel of steroid-hormone signaling but upstream of *daf-12*. One intriguing hypothesis would be that DAU-1 represents a novel inhibitor of the DAF-12 dauer-inducing function and acts independently of steroid-hormones.

### *dau-1* phylogeny indicates multiple gene duplications within the genus *Pristionchus*

Finally, we studied the evolutionary history of *dau-1* in the *Pristionchus* genus. Interestingly, genome-wide analysis revealed that *dau-1* has two additional paralogs in RS2333/California; first, Contig1-snap.329 on chromosome I with 95% amino acid sequence similarity and second, Contig24-snap.126 on chromosome II with 32% amino acid sequence similarity (Fig 4B, S2 Fig). We called these genes *dauerless-like* (*dal*). Phylogenetic analysis indicates that the *dau-1* locus evolved from a *P*. *pacificus*-specific duplication after the split from its sister species *P*. *exspectatus* (Fig 4B). Specifically, *P*. *exspectatus* and *P*. *arcanus* have only one *dal* gene with extremely high sequence similarity to *dau-1* that we named *dal-1* (Fig 4B). All *P*. *pacificus* strains also contain a *dal-1*-like gene. However, the close sequence similarity between *dal-1* of *P*. *exspectatus* and *P*. *arcanus* and *dal-1* and *dau-1* of *P*. *pacificus* make it impossible to determine which gene resulted from the duplication in the *P*. *pacifcus* lineage.

The other paralog of *dau-1*, Contig24-snap.126 is conserved throughout the genus *Pristionchus* and we named this gene *dal-2*. *dal-2* genes were observed in all tested *Pristionchus* species, but they show only limited sequence similarity to *dal-1* and *dau-1*. Furthermore, the only *dal* gene found outside of the genus *Pristionchus* is in the sister genus *Parapristionchus*, whereas *dal* genes were not observed in genomes and genome drafts of 10 more distantly related nematodes, including *C*. *elegans*. Taken together, *dau-1* is an orphan gene that is not found outside *Pristionchus* but has a complex history involving several gene duplications over short evolutionary time scales.

## Discussion

We identified the orphan gene *dau-1* as a regulator of dauer development in *P*. *pacificus* with a potential role in intraspecific competition. Our findings reveal the importance of *dau-1* as a novel or fast-evolving gene with key functions in the dauer regulatory network. Gene duplications and dosage effects by CNV may represent general mechanisms underlying natural variation and our work suggests *daf-12* as the major target for the regulation of dauer development and evolution. Together, our work results in four major conclusions.

### Orphan genes are integral parts of complex regulatory networks

Our work shows that genes that lack high sequence similarity to genes in other species can be of importance for the development, ecology and evolution of an organism. While the conservation of developmental control genes has become a general truism of the modern life sciences [25], only a fraction of all genes in an animal is highly conserved. Genome sequencing projects revealed that a substantial part of genes show limited or no sequence similarity to genes in other organisms [26]. In some cases, such as the sequencing projects of nematodes of 11 different genera, more than 20% of all gene predictions are orphan genes [19]. While transcriptomics and proteomics studies do provide evidence for the expression of orphan genes, little is known about their exact function. This study on *P*. *pacificus dau-1* reveals that orphan genes can indeed be integral parts of more complex regulatory networks, an observation that results in several interesting evolutionary questions.

First, molecular phylogeny of *dau-1* strongly suggests that *dau-1* and related genes evolve rapidly both, with regard to copy numbers and sequence divergence. Therefore, it remains unknown if *dau-1* represents a novel or a fast evolving gene. The fact that the distant paralog Contig24-snap.126 shares only 32% sequence similarity is in line with findings in *Drosophila* that many genes are fast evolving [27,28]. Second, the absence of sequence similarity to genes in other species excludes the usage of gene ontology to obtain first indications for the biochemical function of *dau-1*. Therefore, despite the genetic and molecular evidence for the role of *dau-1* in dauer development as provided in this study, its exact target as inhibitor of dauer development remains currently unknown. We speculate however, that the developmental switch gene *daf-12* is the direct target of *dau-1*, a hypothesis that would be consistent with our epistasis analysis. No matter, if *dau-1* is a novel or fast-evolving gene, its potential interaction with the *daf-12* gene or protein would be intriguing. If *dau-1* is indeed a novel gene, its interaction with DAF-12 would represent a novel inhibitory loop of the dauer regulatory network. Instead, a fast-evolving *dau-1* locus would suggest that parts of the network can evolve rapidly. This would be consistent with the observation that *P*. *pacificus* and *C*. *elegans* DAF-12 show little to no sequence similarity outside of the steroid-ligand and DNA-binding sites although both genes encode for large proteins [24]. While future studies will have to reveal the biochemical function of DAU-1, such work is complicated by the fact that all previous attempts to crystalize the protein have failed so far.

### Dosage effects and a CNV hypothesis for *dau-1* function

**Fig 6:**
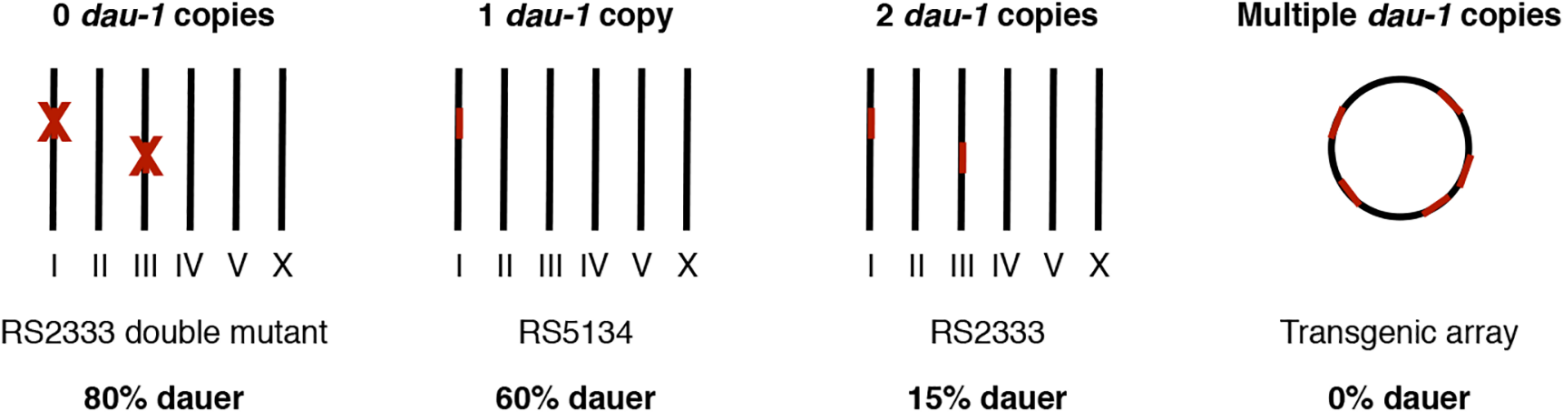
*dau-1* copy number variation. *dau-1* regulates dauer formation and shows a strong dosage effect. One and two copies of *dau-1* result in high and low dauer formation, respectively. *dau-1* double mutants show increased dauer formation, whereas multiple *dau-1* copies inhibit dauer development. Roman numbers represent *P*. *pacificus* chromosomes.

As a second major conclusion, our work supports the general notion, also coming from several studies in medicine, that CNV can affect important developmetal decisions. In the example of *dau-1*, several lines of evidence support the CNV hypothesis (Fig 6): one copy of *dau-1* in the Ohioan strain cause high dauer formation and two copies in the Californian strain causes low dauer formation. In contrast, multiple copies suppress dauer formation altogether, whereas physical and genetic ablation of the CAN neurons and both copies of *dau-1* in RS2333/California result in extremely high dauer formation. Thus, CNV is not only relevant in human disease, but also of importance for invertebrate development. Very recent genomic studies further support the notion that CNV represents a widespread evolutionary phenomenon. The comparison of young genes across multiple stickleback populations revealed extensive CNV again linking CNV with fast evolving genes [29].

In retrospect, we were surprised that CNV of *dau-1* resulted in a mappable QTL on chromosome I, which harbors a copy in both strains, RS2333/California and RS5134/Ohio. Whereas the lower recombinantion frequency in the concerned area of chromosome III prevented us from identifying the associated gene, which might well be *dau-1*.*2*, we assume that differences in the expression and/or activity of *dau-1*.*1* between RS2333/California and RS5134/Ohio are responsible for the QTL associated with ME25944. Indeed, *dau-1*.*1* has 5 SNPs resulting in amino acid differences between DAU-1.1 of RS2333/California and RS5134/Ohio and a total of 13 SNPs in the duplicated area. However, the strong effects observed after *dau-1* overexpression in transgenic animals in any combination (Table 1 c-g) prevents us from using swapping experiments to investigate the role of individual SNPs.

The presence of additional *dal* genes in the *P*. *pacificus* genome results in specific questions that will be addressed in future studies. CRISPR-Cas9 induced gene inactivation can be used to study the function of Contig1-snap.329 and Contig24-snap.126, but first attempts to produce such mutants failed (M.G.M., J. deVriend, M. Atzhigi & R.J.S.). One intriguing hypothesis would be that *dal-1*, the gene with the highest sequence similarity to *dau-1*.*1* and *dau-1*.*2*, is also involved in the inhibition of dauer development. In this context it is important to note that even the double knockout phenotype of *dau-1*.*1* and *dau-1*.*2* (Table 1 x) does still not result in 100% dauer formation, a function that might well be attributed to *dal-1*. If so, it is interesting to note that such a potential function of *dal-1* in *P*. *pacificus* would be independent of the CAN neurons, as *dau-1*.*1; dau-1*.*2* double mutants completely mimic the CAN ablation phenotype.

### The CAN neurons as novel cells in dauer regulation

Our results provide novel aspects of the cellular and genetic mechanisms of nematode dauer development. Besides the identification of *dau-1*, we show a role of the CAN neuron in the regulation of *P*. *pacificus* dauer formation. The *P*. *pacificus* CAN neuron is similar to the corresponding cell in *C*. *elegans*, both by position and form. However, the function of both cells differ: in *C*. *elegans* the CAN neuron is essential for viability and CAN ablation results in the death of the larvae suggesting a function in osmoregulation. In *P*. *pacificus*, CAN ablation is viable facilitating the identification of its role in dauer regulation. Thus, our work reveals new functions for a new gene, *dau-1*, and a new function for a previously unconsidered cell. Additional studies are necessary to link the *dau-1* expression pattern to other genetic components of the regulatory network. Unfortunately, the *P*. *pacificus* transgenic system only allows the introduction of DNA fragments smaller than 20 kb [30], preventing us from studying the expression pattern of the *Ppa-daf-12* locus, which is more than 40 kb in size.

### The role of *dau-1* in the regulation of intraspecific competition

Our work on *dau-1* provides a molecular mechanism for cross-preference and intraspecific competition among *P*. *pacificus* strains. While previous studies already indicated strong natural variation in pheromone production and sensing supporting intraspecific competition as a new role in nematode ecology, these studies did not investigate the molecular mechanisms involved in the integration of upstream pheromone variation into the dauer regulatory network [11,12]. The results of our epistasis analysis clearly indicate that *dau-1* acts downstream of small-molecule pheromones at the level of endocrine hormone signaling, which is known as convergence point of various signaling inputs in *C*. *elegans* dauer regulation. Therefore, the regulation of *daf-12* emerges as a key principle in dauer development and evolution. In this context it is important to note that *daf-12* is the key developmental switch gene for the dauer *vs*. direct developmental decision and such developmental switch genes have long been predicted to represent major features of phenotypic plasticity in animals and plants [31].

Finally, while our work starts to identify the molecular mechanisms associated with natural variation and intraspecific competition between *P*. *pacificus* strains, this work does not touch on the ecological implications. We have previously shown that multiple, distinct *P*. *pacificus* haplotypes can be found on the same living beetle [4] and that even closely related strains can differ tremendously in dauer longevity and fitness [13]. The majority of the available more than 800 wild isolates of *P*. *pacificus* have been sampled as dauer stages from scarab beetles and all tested strains can still form dauers indicating that no strains represents a Daf-d phenotype. RS5134/Ohio represents an extreme example with high dauer induction, high dauer survival and relatively high fitness after dauer recovery, whereas RS2333/California shows much lower dauer induction, survival and recovery (Fig 1). In light of our molecular findings on *dau-1*, these life history traits result in several ecological conclusions and questions. *dau-1* cannot be the only molecular player involved in the observed natural variation pattern between strains, as only RS2333/California and RS106/Poland have the additional *dau-1* copy. Also, the majority of strains have only one *dau-1* copy but still differ in dauer induction and longevity indicating that additional factors must exist. In particular, molecular differences are to be expected in dauer physiology, a phenomenon little studied in both *C*. *elegans* and *P*. *pacificus* [20]. Future studies can adress differences in dauer physiology using transcriptomic approaches between strains and might identify mechanisms involved in the different survival pattern of nematode strains.

## Materials and Methods

### Nematode cultures

Nematodes were grown on nematode growth medium (NGM) agar plates with the *E*. *coli* strain OP50 as food source [32]. Crosses were performed using one J4 hermaphrodite and two males.

### Dauer pheromone assays

Dauer pheromone was purified from nematode liquid cultures as described previously [13]. The dauer pheromone assay was modified for three different types of experiments. First, for assays with transgenic animals (Table 1 a-h), a mixture of 190 μl water and 10 μl pheromone was distributed evenly over the surface of NGM agar plates. We spotted each plate with 20 μl kanamycin-treated OP50 [11]. Per plate, four young adult hermaphrodites were allowed to lay eggs overnight, producing approximately 100 progeny. After three days, dauer formation was calculated as the percentage of progeny that entered the dauer stage. Second, epistasis experiments with transgenic animals (Table 1 i-p) were performed as described above but using NGM agar plates lacking cholesterol. Third, to combine ablation experiments with dauer pheromone assays, the assay was modified to enable the use of J2 larvae instead of adults. The dauer formation of mutant animals was also tested using J2 larvae to obtain comparable results. For assays with ablated and mutant animals (Table 1 q-z), a mixture of 180 μl water and 20 μl pheromone was distributed evenly over the surface of NGM agar plates lacking cholesterol. We spotted 15 μl kanamycin-treated OP50 and picked 50 J2 larvae onto each plate. After two days, dauer formation was calculated as the percentage of J2 larvae that entered the dauer stage. To test if the *dau-1*.*1;dau-1*.*2* double mutant responds to DA, 15 μl of ∆7-DA were added to each plate, resulting in a final concentration of 15 μM. The mean dauer formation of three independent biological replicates was calculated for all experiments. No dauers were observed on control plates without pheromone.

### Competition assays

Competition assays were performed in Ussing chambers without adding external dauer pheromone [11]. For the competition experiment, RS2333/California was grown in liquid culture in one compartment of an Ussing chamber, while RS5134/Ohio was grown in the other compartment of the same chamber. As controls, a second chamber contained only RS2333/California in both compartments, and a third chamber contained only RS5134/Ohio. At the beginning of the assay, the nematodes from one fully- grown NGM agar plate, initially containing 10 J4 larvae, were washed off into one compartment. For each time point (7, 8, 9, 10, 11, 12, 13, and 14 days), three replicates of a sample volume of 30 μl were taken from each compartment, and dauer formation was calculated as the percentage of dauers in the sample volume. The mean dauer formation of the three replicates was calculated for each time point. We repeated all experiments multiple times and obtained similar results.

### RILs and QTL mapping

After crossing RS2333/California males with RS5134/Ohio, heterozygous F1 animals were inbred for 10 generations to generate 911 RILs (S1A Fig). For each RIL, we determined the dauer formation phenotype in response to both parental pheromones. We selected 136 RILs with low, medium, and high dauer formation phenotypes for genotyping with simple sequence length and conformation polymorphism markers (S1 Table). QTL mapping was performed using the qtl package of the program R [33] (S1B Fig).

### Microinjection and transgenic lines

Transgenic lines were generated as described previously [30]. A 9.5 kb genomic RS2333/California or RS5134/Ohio construct, consisting of a 3.3 kb upstream region, the 1.7 kb *dau-1* gene, and a 4.5 kb downstream region, was injected into RS2333/California or RS5134/Ohio animals. To generate reporter lines, an RS2333/California translational reporter construct, containing a 4.7 kb upstream region and the *dau-1* coding region driving RFP expression, was injected into RS2333/California animals. We obtained three independent reporter lines. Non- transgenic nematodes that had lost the transgenic array were used as controls in dauer pheromone assays. A RS2333/California line expressing only the RFP injection marker was generated as an additional control.

### Ablation experiments

Cell ablation was performed as described previously [34]. The CAN neurons were ablated in freshly hatched J2 larvae, which were then immediately transferred to dauer pheromone assay plates. After determining the dauer formation phenotype, animals were checked to confirm the absence of CAN neurons. Nematodes, in which the CAN neurons were still present, were excluded from the calculation of the mean dauer formation of the three biological replicates.

### CRISPR/Cas9 system

CRISPR/Cas9 induced gene inactivation was performed as described previously [23]. After injection, 576 F1 animals were screened for deletions by Sanger sequencing. We obtained two mutant lines (*tu490* and *tu491*) with a 7 bp and a 5 bp deletion in *dau-1*.*1* and one mutant line (*tu492*) with a 10 bp deletion in *dau-1*.*2*. Deletion mutants were backcrossed multiple times, and double and triple mutants were generated by Mendelian genetics.

### Computational and phylogenetic analyses

To analyze the genomic region associated with the QTL ME25944 for SNPs, deletions, and duplications, we used the genomic resequencing data from 104 *P*. *pacificus* strains [22]. The *dau-1* locus was identified as being duplicated in RS2333/California when compared to RS5134/Ohio by the software cnv-seq based on a significant difference in read coverage (*P*<10^–39^) [35]. For comparisons of gene expression levels, we prepared and analyzed RNA-seq libraries as described previously [36]. Quantification of expression levels as fragments per kilobase transcripts per million fragments sequenced showed strong similarity between the two strains (Spearman's ρ = 0.75, *P*<10^−16^). 1298 genes were identified as being differentially expressed (FDR<0.05) [37], among which we found the gene prediction corresponding to *dau-1*.*1* (Contig44- snap.18, version Hybrid1). To investigate the evolutionary history of *dau-1*, we reconstructed maximum likelihood trees using homologous sequences from other diplogastrid nematodes: gene predictions for *P*. *exspectatus* [22], gene predictions for *P*. *arcanus*, and a transcriptome assembly for *Parapristionchus giblindavisi*. Protein sequences were aligned using the MUSCLE aligner (version v3.8.31) [38], and maximum likelihood trees were calculated using the phangorn R package [39] after selection of the best model using the ProtTest3 webserver [40].

### Statistical analyses

95% confidence intervals were calculated for the mean dauer formation values obtained in dauer pheromone assays and competition assays. To compare dauer formation of two strains, Fisher's exact test was performed using the program R (www.r-project.org), and *p* values less than 0.05 were considered statistically significant.

## Supporting Information

S1 Fig RILs and QTL analysis. (**A**) Crossing scheme resulting in 911 RILs. (**B**) QTL peaks with significant LOD scores. The red line represents the significance threshold. We obtained six significant QTL peaks for dauer formation in response to the RS5134 pheromone, four of which are also significant for dauer formation in response to the RS2333 pheromone. Fine mapping enabled us to narrow down the QTL peak associated with the marker ME25944 (indicated by a red *). ME25944 was chosen for being the peak with the highest LOD score in response to the RS5134 pheromone and because our attempts to narrow down the regions associated with the other QTL peaks failed for molecular reasons.
(PDF)

S2 Fig Multiple sequence alignment. Alignment of the predicted protein sequences of *Pristionchus* and *Parapristionchus dau-1* homologs.
(PDF)

S1 Table Phenotypes and genotypes of 136 RILs used for QTL analysis. (XLSX)
