# Supplementary figures and images for: The Orphan Gene dauerless Regulates Dauer Development and Intraspecific Competition in Nematodes by Copy Number Variation
Source: PLoS Genet. 2015 Jun 18;11(6):e1005146. doi: 10.1371/journal.pgen.1005146 (PMC4473527; doi:10.1371/journal.pgen.1005146)

**A**

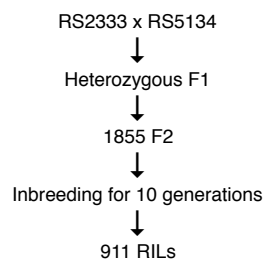

**B**

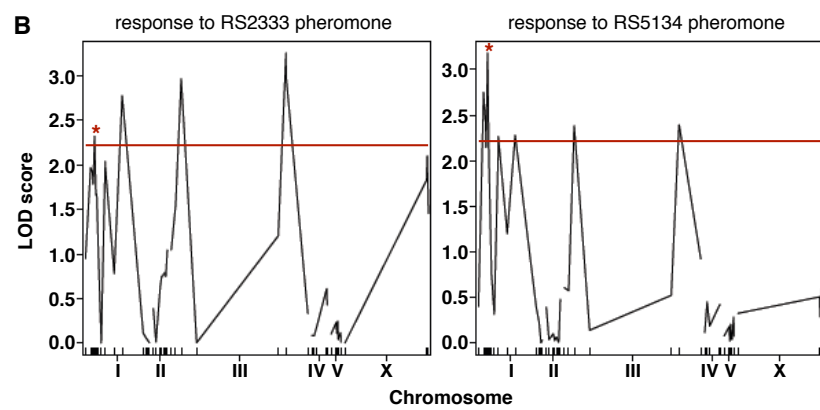

Supplement: S1 Fig — (A) Crossing scheme resulting in 911 RILs. (B) QTL peaks with significant LOD scores. The red line represents the significance threshold. We obtained six significant QTL peaks for dauer formation in response to the RS5134 pheromone, four of which are also significant for dauer formation in response to the RS2333 pheromone. Fine mapping enabled us to narrow down the QTL peak associated with the marker ME25944 (indicated by a red *). ME25944 was chosen for being the peak with the highest LOD score in response to the RS5134 pheromone and because our attempts to narrow down the regions associated with the other QTL peaks failed for molecular reasons. (PDF) [file pgen.1005146.s001.pdf]
