# Supplementary material for: The Orphan Gene dauerless Regulates Dauer Development and Intraspecific Competition in Nematodes by Copy Number Variation
Source: PLoS Genet. 2015 Jun 18;11(6):e1005146. doi: 10.1371/journal.pgen.1005146 (PMC4473527; doi:10.1371/journal.pgen.1005146)
Supplement: S2 Fig — Alignment of the predicted protein sequences of Pristionchus and Parapristionchus dau-1 homologs. (PDF) [file pgen.1005146.s002.pdf]

```
RS5555_Locus_10591_isoform_1          --------LRMSLTPTVTERKQIALLLLAGEQYDCYK-HLLNDDRAEEMNTFQVFNRIME
RS5527_scaffold251-parc_tblastn       MSPRHCLSSSICFSPDAFDRRIISLLIVAGEQFEEFKVRVLGADSK--DTIYSAIHIARQ
RS5522_scaffold260-EXSNAP2012.13      MSSRLCLSSSISFSPDVFDRRVIALLIVAGEQFEEFKSSVLGVDSK--DTIYSAIDLARR
RS2333_Contig1-snap.329               MSSRHWLPSSIRYSPDAFDRRIIALLIVAGEQFEEFKSSVLGDDSK--DTIYSAIDLARR
RS2333_Contig44-snap.18_RACE          MSSRHWLPFSIRYSPDAFDRRIIALLIVAGEQFEEFKSSVLGADSK--DTIYSAIDLARR
RS5134_Contig44-snap.18_RACE          MSSRHWLPFSIRYSPDAFDRRIIALLIVAGEQFEEFKSSVLGDDSK--DTIYSAIDLARR
RS5522_scaffold1181-EXSNAP2012.3      MASR-TVSVVTRVSPDADERQIIALLMLTGQQFDEFKTLLLSNSKIHFENVFTAANTLRR
RS2333_Contig24-snap.126              MASR-TNSRVTRVAPNAHERQVIALLMLTGQQFDEFKILLFTNPKHHVENVFTAARTLQK
RS5527_scaffold_5-parc_aug2013-1      MASR-AISCLTHVSPTAYDRQLIALLMLTGQQFDEFKDLLLYNMNS--ETVFTAANTLQK
                                                   :* . :*. *:**:::*:*:: :*  ::        . : .

RS5555_Locus_10591_isoform_1          YGRLSQFVQPKNRPLVLSLALNYERQLAKLGRSLGSRRLEVSDSV----------CKSDY
RS5527_scaffold251-parc_tblastn       CLTNADYVPESQWPLVIQIVRCFQSQFEKLIKSIGEEKVDDYAGLQSNELVNSRISMDKY
RS5522_scaffold260-EXSNAP2012.13      FLATADYVPESQWPFVIQIVRCYQSHFEKLIKSIGEEKVDDYAGLQSSQLFNSRISMDMY
RS2333_Contig1-snap.329               FLATADYVPESQWPLVIQIIRCYQSQFEKLIRSIGEEKVDDYAGLQSNELFNSRISMDKY
RS2333_Contig44-snap.18_RACE          YLATADYVPESQWPLVIQIIRCYQSQFEKLIRSIGEEKVDDYAGLQSNELFNSRTSMDKY
RS5134_Contig44-snap.18_RACE          YLATADYVPESQWPLVIQIIRCYQSQFEKLIRSIGEEKVDDYAGLQSNELFNSRTSMDKY
RS5522_scaffold1181-EXSNAP2012.3      YLSRADYVPPNERCLVMEIALMYESQLKKLIAEIGESVVNSIEEI--NDIYDQ--TYEKH
RS2333_Contig24-snap.126              YLALADCVPCNKRCMVMEIALMYESQLKKLIAELGESVMDSIEEI--NYVNEQ--TRERY
RS5527_scaffold_5-parc_aug2013-1      YLALADYVPSNKRCLVMEIALLYESQFKKLISELGESVVESIEEI--NDVNDQ--TREKY
                                          :: *  .:. :*:.:   :: :: **  .:*.  ::    :            . :

RS5555_Locus_10591_isoform_1          GKLNWKFFRIETPPMTTTIFHD--RIPMVKQSLGEIVLAKAIQCFTESNLPS-VPIPPQK
RS5527_scaffold251-parc_tblastn       LVKKS----VQVGDFITTTFPPAIRRPFGKVD-EFLVKKSVVDIISQPNLKV-IETLPIT
RS5522_scaffold260-EXSNAP2012.13      F--------VQVGDFITTMFPPAIRRPFGKAD-AFLIKKS-VDVLSQLNQKV-IESQPIA
RS2333_Contig1-snap.329               S--------VQIGDFITTMFPPAIRRPFSKVD-AFLIKKS-VDLLSEPNLKV-IESQTNA
RS2333_Contig44-snap.18_RACE          S--------VQIGDFITTMFPPAIRRPFSKVD-AFLIKNS-VDLLSEPNLKV-IESQAIA
RS5134_Contig44-snap.18_RACE          S--------VQIGDFITTMFPPAIRRPFSKVE-AFLIKNS-VDLLSEPNLKV-IESQAIA
RS5522_scaffold1181-EXSNAP2012.3      QNRNW-----VKCKLMHTIFRTGSRKPIEKGTLSYLALKSAMLALSQPNNKVEMVLIDDE
RS2333_Contig24-snap.126              LNRNW----AVECNFMHTIFRTGSRKPIEKGTLSYLTLKSAALALSQPNHEE-FELIDEQ
RS5527_scaffold_5-parc_aug2013-1      LNRNW----AKECNLMHTIFRTGSRKPIEKGSLSYLALESAVFALSQPNYEE-FVLIDEQ
                                                    :  * *    * *: *     :   .    ::: *    .

RS5555_Locus_10591_isoform_1          DSNRTKEDPCYGNRKRIFPSRKRRLSMVELEAPCAKRAARHEDTDEAREMSN--NDWIME
RS5527_scaffold251-parc_tblastn       KIQASEE-PALLSRPQMRFARPRRISMSPIYETFEKGL--KTIKEEADFEENNFNDELTN
RS5522_scaffold260-EXSNAP2012.13      KIQSTEE-PVLLSRPQLRFARPRRISMSPIYETCEKGLFFKTIKVEADFEENNFNDELTN
RS2333_Contig1-snap.329               KPQATEE-PALLFRPQQRFARPRRISMSPIYETCEKGLFFKTIKVEADFEENNFNDELTN
RS2333_Contig44-snap.18_RACE          KPQATEE-PALLSRPQQRFSRPRRISMSPIYETCEKGLFFKTIKVEADFEENNLYDELTN
RS5134_Contig44-snap.18_RACE          KPQATEE-PALLSRPQQRFARPRRISMSPIYETCEKGLFFKTIKVEADFEENNLYDELTN
RS5522_scaffold1181-EXSNAP2012.3      PWNTHKEEPTLPTVPIIRPPRKRRYSMVSTEEFASKRLRTEADKEAMRAVAVEENFYVGD
RS2333_Contig24-snap.126              TWISQKEEPTLPTAPIINPPRKRRNSMVLTEEYPVKRLRTNGEKEAMRAMAVEDNTYVHD
RS5527_scaffold_5-parc_aug2013-1      AWYSHKEEPTLPTVPIIRPPRKRRCSMISTEEFAAKRLRTNAEKAAMRALADEDNSHIHD
                                           :* *          .* ** **        *       .             : :

RS5555_Locus_10591_isoform_1          DEA--FIDYEIDQFKDYC--RNSDVPIIERWLCSRSLR--------IHGTETHPDCESRI
RS5527_scaffold251-parc_tblastn       NIAFQFQDHEVSEFT-FC--EKTAVPAKERFDCSSALFDWN----YTHNGGSHYCCRQSA
RS5522_scaffold260-EXSNAP2012.13      NVA--FQDHEVTEFA-FC--EKINASARERFICASALFDCN----YTHNGGSHHDCRQSA
RS2333_Contig1-snap.329               NIA--FQDHEVTEIA-FC--EKINASARERFSCASDLFDCI----YTDNGGSHYDC----
RS2333_Contig44-snap.18_RACE          NVA--FKDHEVTEFA-FC--EKINASARERFSCASALFDCI----YTDNGGSHYDCRQSA
RS5134_Contig44-snap.18_RACE          NIA--FKDHEVTEFA-FC--EKINASARERFSCASALFDCI----YTDNGGSHYDCRQSA
RS5522_scaffold1181-EXSNAP2012.3      DIA--FEETIVPQFIHYCRAHTKYASNAERWACSRNLRNHLTRNEEADESAIHEKCLEET
RS2333_Contig24-snap.126              DIA--FEETILPRFIHYCRADTAETSNGERHN----------------------------
RS5527_scaffold_5-parc_aug2013-1      DIA--FEETIMPRFIHYCRTDAKDASNGERWACSRNLRNRLTRNEVANESEIHEKCLEDT
                                      : *  * :  :  :  :*      ..  **

RS5555_Locus_10591_isoform_1          RNDRSSEPDMSYVALKQR-PAKFSREFDPVLPSRHRSFFH-
RS5527_scaffold251-parc_tblastn       MDRKA-TIENSGRIIDGK-STK--REFEFVTKQIKVMYLHE
RS5522_scaffold260-EXSNAP2012.13      MDRKTWKCDSH------------------------------
RS2333_Contig1-snap.329               ------LCKSHFHIV----------VVYYLSQLKIVADCSE
RS2333_Contig44-snap.18_RACE          MDRKA-SSENSSRIIDGK-STK--REFEFINKHMKVMYLHE
RS5134_Contig44-snap.18_RACE          MDRKA-SSENSSRIIDGK-STK--REFEFINKHMKVMYLHE
RS5522_scaffold1181-EXSNAP2012.3      FNDQSYFLAIRSSQKQSK-VLS-------------------
RS2333_Contig24-snap.126              -----------------------------------------
RS5527_scaffold_5-parc_aug2013-1      MRDRSYFLAIRSSQVNKRYNQQFYREFWPIVPFENSSNLV-
```

Multiple sequence alignment (MUSCLE (3.8)) of *Pristionchus* and *Parapristionchus* dau-1 homologs.
